# Stem cell-derived tissue- associated regulatory T cells ameliorate the development of autoimmune arthritis

**DOI:** 10.1186/2051-1426-3-S2-P21

**Published:** 2015-11-04

**Authors:** Mohammad Haque, Kristin Fino, Jianxun Song

**Affiliations:** 1 Pennsylvania State University College of Medicine, Hershey, PA, USA

## Background

Embryonic stem cells have the ability to grow indefinitely while maintaining pluripotency. Under the right circumstance, pluripotent stem cells (PSC) can produce almost all of the cells in the body including regulatory T cell (T_regs_). T_regs_ are essential for normal immune surveillance systems, and their dysfunction leads to the development of diseases, such as autoimmune disorders. PSC are a potential renewable source of healthy T_regs_, which could treat a wide array of autoimmune disorders. However, the right circumstances for the development of antigen (Ag)-specific T_regs_ from PSC (*i.e.*, PSC-T_regs_) has not been well defined. So the purpose of this study is to develop antigen-specific T_regs_ from pluripotent stem cells for treatment of Ag-induced arthritis (AIA) in mice.

## Methods

In this study mouse iPS cells were transduced with FoxP3 and ovalbumin-specific TCR (OTII). iPS cells were driven toward T lymphocyte lineage by co-culture on OP9 cells expressing delta-like (DL1), a Notch ligand. The combination of Notch signaling, FoxP3, and TCR drove iPS cell to differentiation into Ag-specific T_regs_. In vitro generated T_regs_ were adoptively transferred into AIA mice.

## Results

Our results showed that TCR transduced iPS cells differentiated into T_regs_ and express CD3, TCRβ, CD4, CD8, CD25, CTLA4 *in vitro*. Adoptive transfer of such T_regs_ dramatically suppressed autoimmunity in a well-established AIA model, including the inflammation, joint destruction, cartilage prostaglandin depletion, osteoclast activity, and Th17 production.

**Figure 1:**
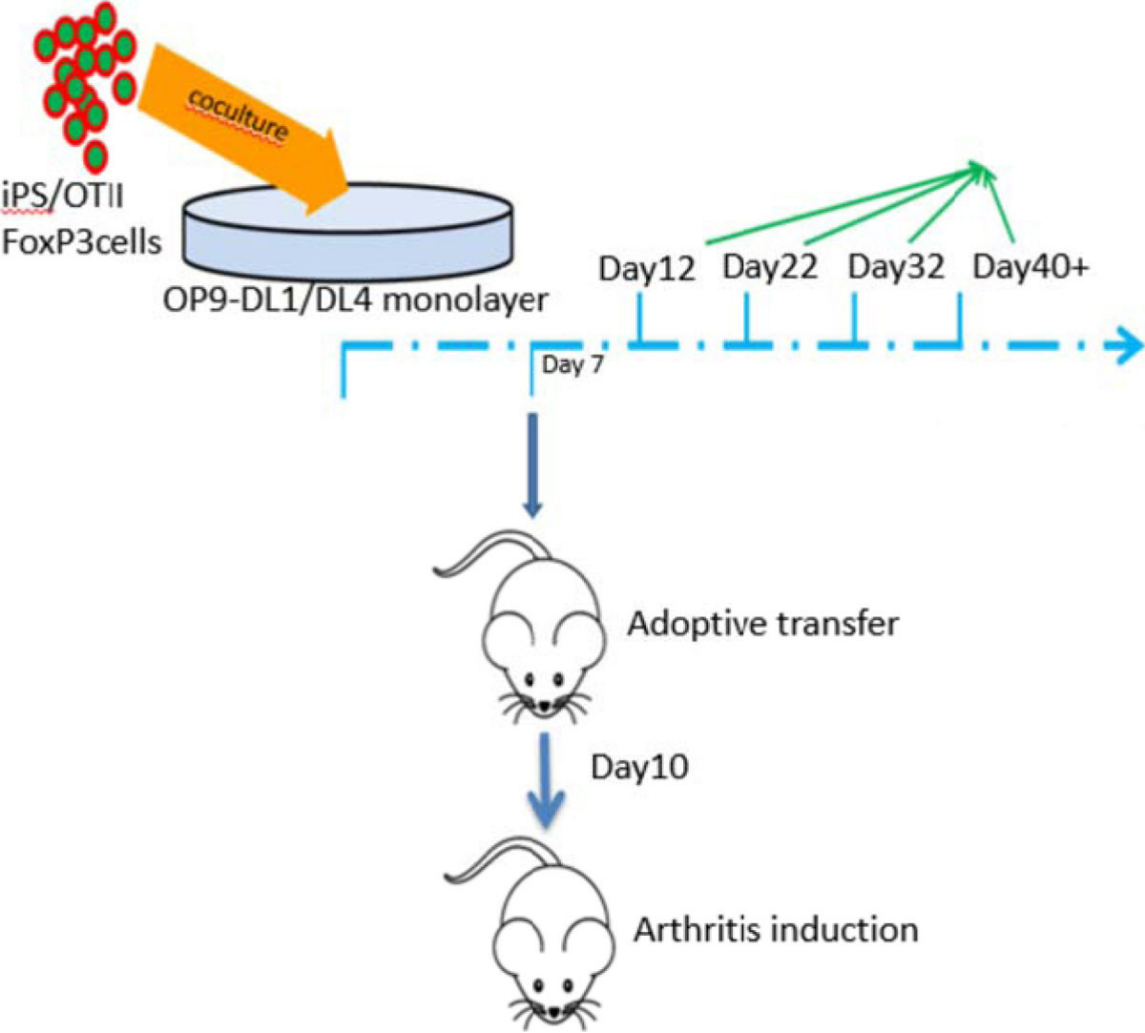
Model of the experiment.

**Figure 2:**
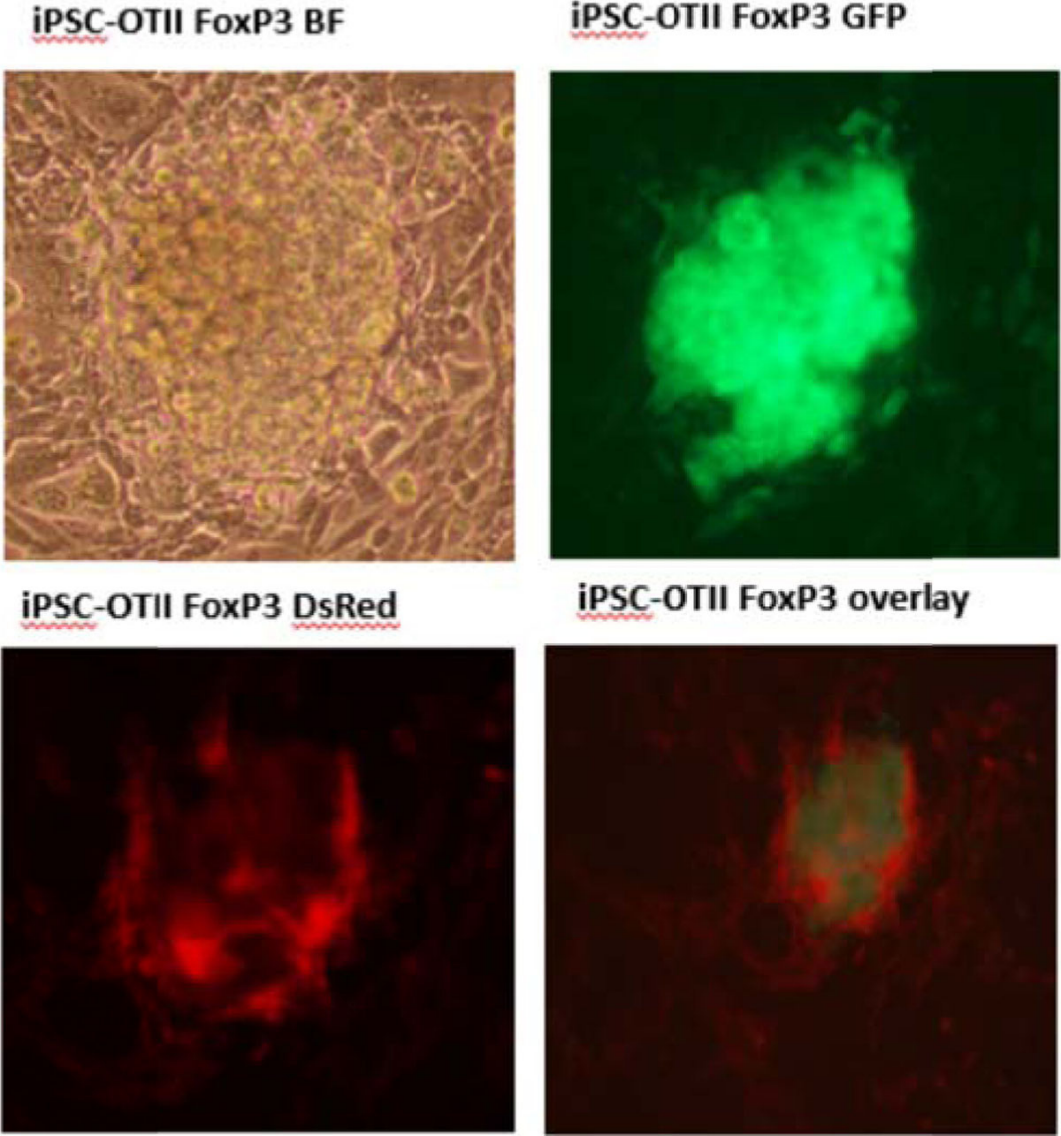
Retroviral transduction result.

**Figure 3:**
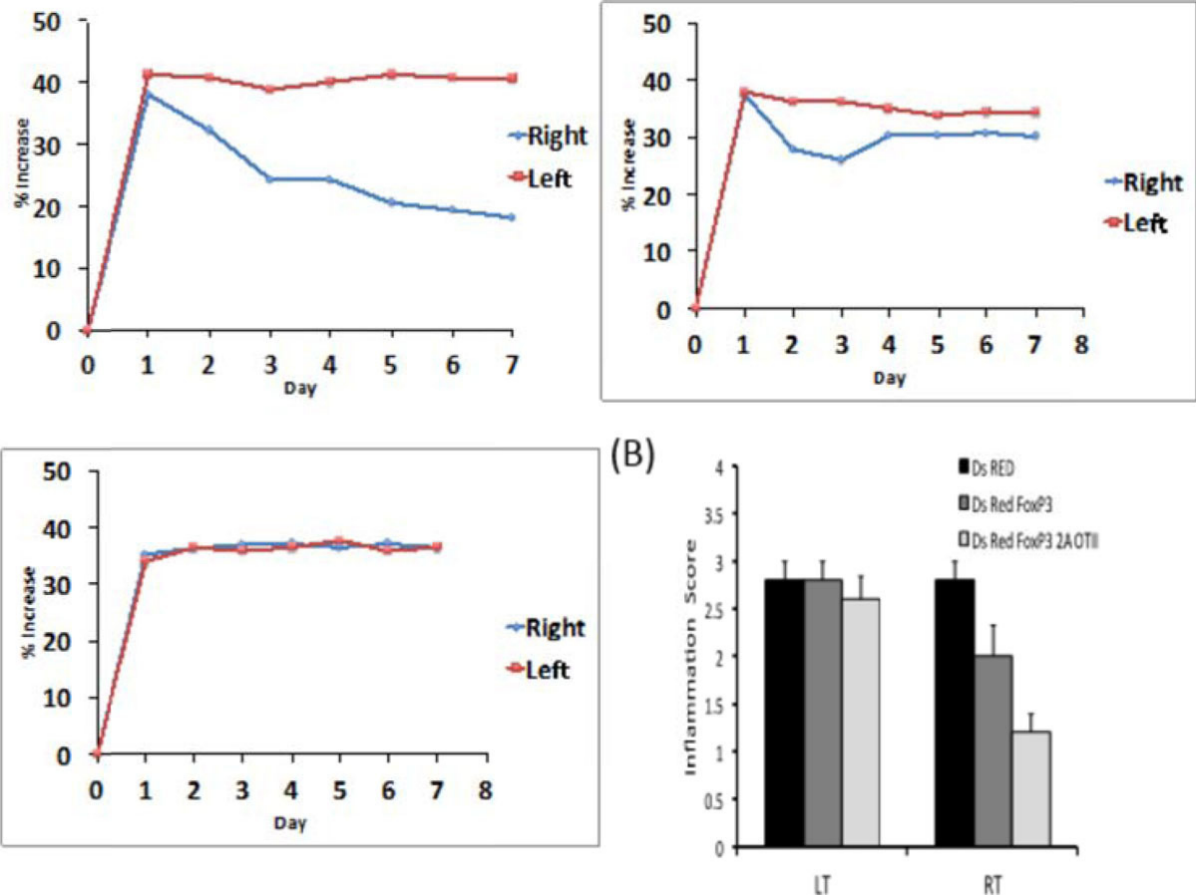
Arthritis development was reduced in adoptively transferred mice.

**Figure 4:**
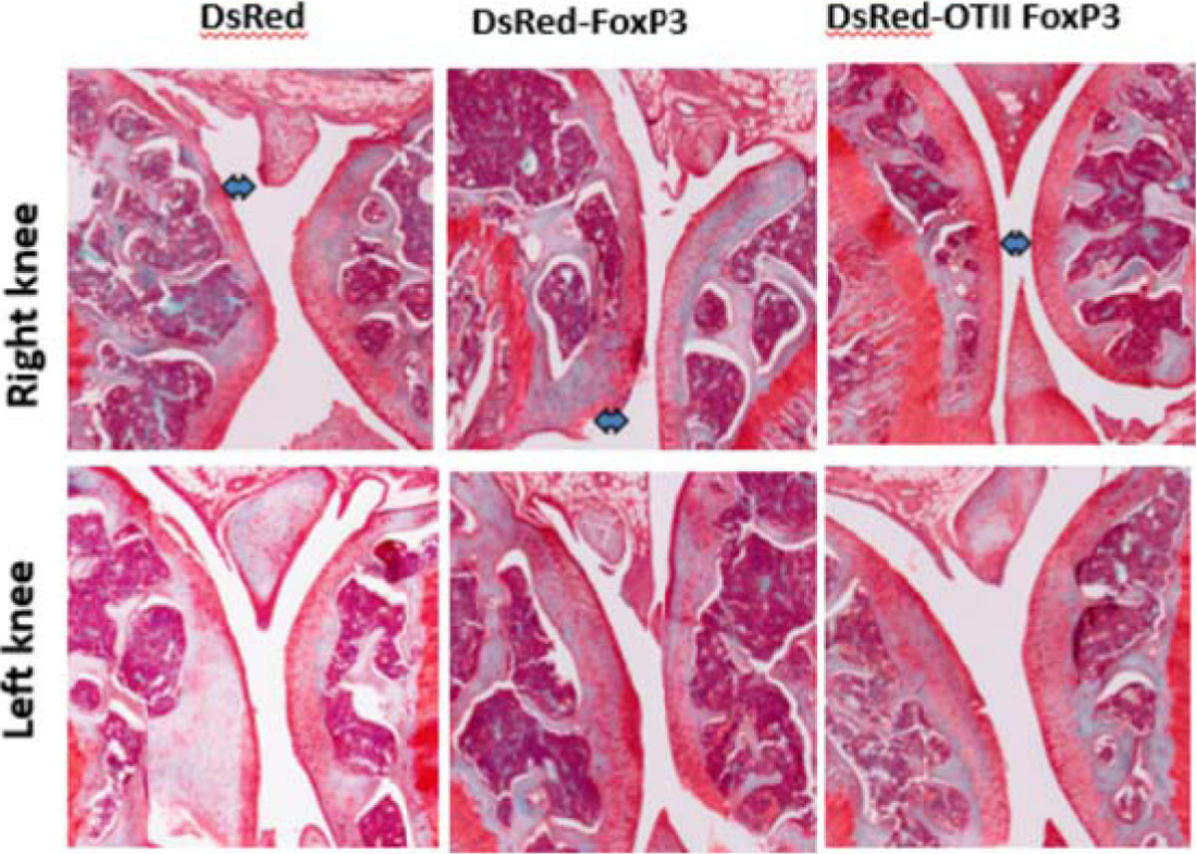
Joint destruction.

**Figure 5:**
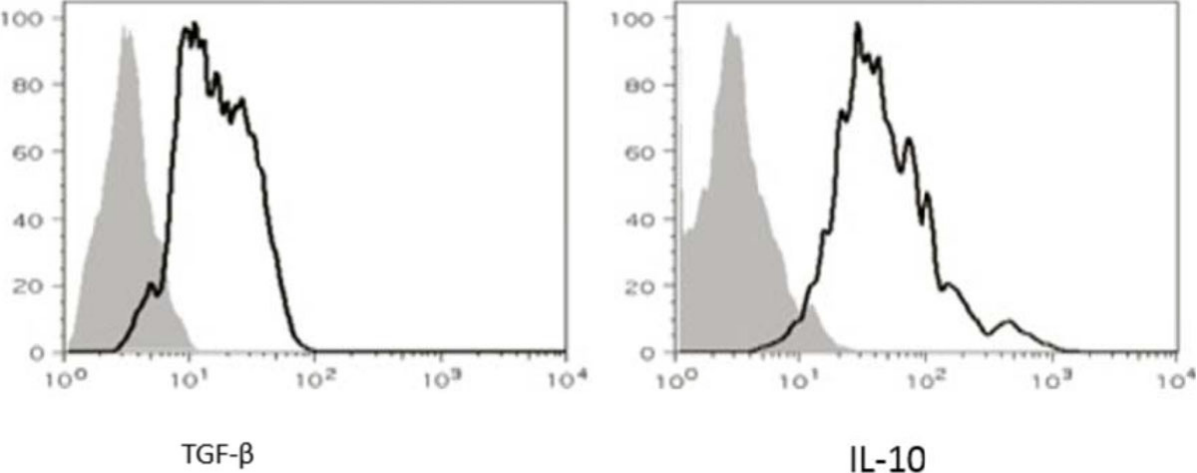
Adoptively transferred cells are functional.

**Figure 6:**
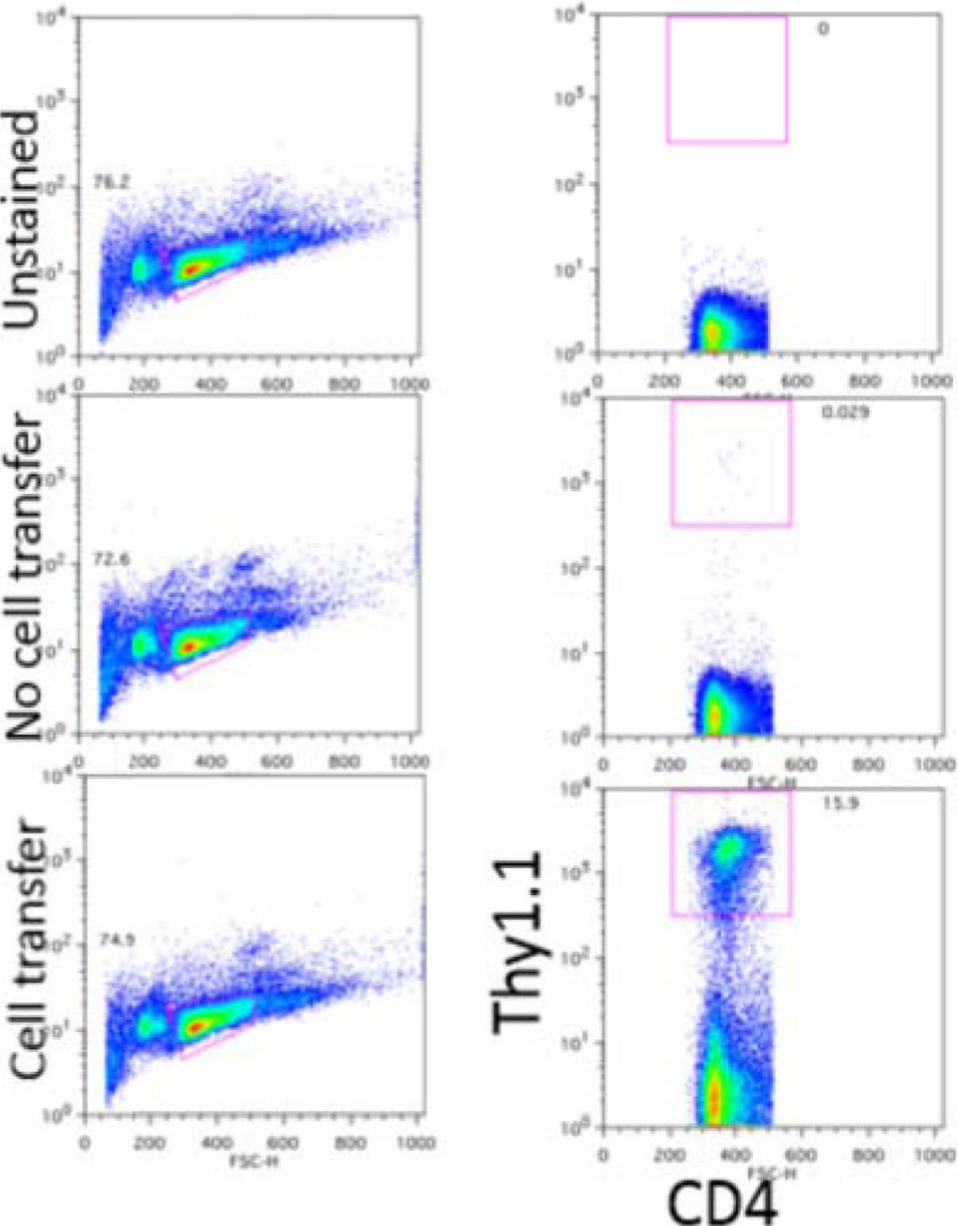
Adoptively transfer cells are in persistence in vivo.

